# Metabolomic profiling reveals decreased serum cysteine levels during gestational diabetes mellitus progression

**DOI:** 10.1093/jmcb/mjae010

**Published:** 2024-03-01

**Authors:** Mengyu Lai, Jiaomeng Li, Jiaying Yang, Qingli Zhang, Yujia Gong, Yuhang Ma, Fang Fang, Na Li, Yingxiang Zhai, Tingting Shen, Yongde Peng, Jia Liu, Yufan Wang

**Affiliations:** Department of Endocrinology and Metabolism, Shanghai General Hospital, Shanghai Jiao Tong University School of Medicine, Shanghai 200080, China; Shanghai Institute of Materia Medica, Chinese Academy of Sciences, Shanghai 201203, China; Department of Endocrinology and Metabolism, Shanghai General Hospital, Shanghai Jiao Tong University School of Medicine, Shanghai 200080, China; Shanghai Institute of Materia Medica, Chinese Academy of Sciences, Shanghai 201203, China; Department of Endocrinology and Metabolism, Shanghai General Hospital, Shanghai Jiao Tong University School of Medicine, Shanghai 200080, China; Department of Endocrinology and Metabolism, Shanghai General Hospital, Shanghai Jiao Tong University School of Medicine, Shanghai 200080, China; Department of Endocrinology and Metabolism, Shanghai General Hospital, Shanghai Jiao Tong University School of Medicine, Shanghai 200080, China; Department of Endocrinology and Metabolism, Shanghai General Hospital, Shanghai Jiao Tong University School of Medicine, Shanghai 200080, China; Shanghai Institute of Materia Medica, Chinese Academy of Sciences, Shanghai 201203, China; School of Pharmacy, Henan University, Kaifeng 475004, China; Department of Endocrinology and Metabolism, Shanghai General Hospital, Shanghai Jiao Tong University School of Medicine, Shanghai 200080, China; Department of Endocrinology and Metabolism, Shanghai General Hospital, Shanghai Jiao Tong University School of Medicine, Shanghai 200080, China; Shanghai Institute of Materia Medica, Chinese Academy of Sciences, Shanghai 201203, China; School of Pharmaceutical Science and Technology, Hangzhou Institute for Advanced Study, University of Chinese Academy of Sciences, Hangzhou 310058, China; Department of Endocrinology and Metabolism, Shanghai General Hospital, Shanghai Jiao Tong University School of Medicine, Shanghai 200080, China

**Keywords:** gestational diabetes mellitus, metabolomics, cysteine

## Abstract

Gestational diabetes mellitus (GDM) is a pregnancy-related metabolic disorder associated with short-term and long-term adverse health outcomes, but its pathogenesis has not been clearly elucidated. Investigations of the dynamic changes in metabolomic markers in different trimesters may reveal the underlying pathophysiology of GDM progression. Therefore, in the present study, we analysed the metabolic profiles of 75 women with GDM and 75 women with normal glucose tolerance throughout the three trimesters. We found that the variation trends of 38 metabolites were significantly changed during GDM development. Specifically, longitudinal analyses revealed that cysteine (Cys) levels significantly decreased over the course of GDM progression. Further study showed that Cys alleviated GDM in female mice at gestational day 14.5, possibly by inhibiting phosphoenolpyruvate carboxykinase to suppress hepatic gluconeogenesis. Taken together, these findings suggest that the Cys metabolism pathway might play a crucial role in GDM and Cys supplementation represents a potential new treatment strategy for GDM patients.

## Introduction

Gestational diabetes mellitus (GDM) is a pregnancy-related metabolic disorder affecting 9%–26% of pregnant women in various countries, and its prevalence has been increasing in recent decades (Johns et al., 2018). GDM usually presents as mild hyperglycemia during pregnancy and leads to adverse pregnancy outcomes, including gestational hypertension, pre-eclampsia, and polyhydramnios. For fetuses and neonates, macrosomia, preterm birth, birth injury, shoulder dystocia, neonatal hypoglycemia, and respiratory distress may occur (Flenady, 2008). Furthermore, GDM increases the risk of developing type 2 diabetes mellitus (T2DM) and cardiovascular disease in the long term (Daly et al., 2018). Although increased insulin resistance and inadequate compensation for the body's insulin needs are well established to be associated with GDM, the detailed mechanism has not been fully characterized (Catalano, 2014). Therefore, it is imperative to elucidate the underlying mechanisms of the physiological transition from normal glucose tolerance (NGT) to GDM.

Investigating metabolic profiles can provide novel and deeper insights into the etiopathogenesis of diseases (Johnson et al., 2016). The metabolic profiles of women with GDM revealed significant alterations in specific metabolites, including lipids (phosphatidylcholines [PCs], sphingomyelins [SMs], and triglycerides [TGs]), carbohydrates (glucose and fructose), and amino acids (AA; branched-chain AA and aromatic AA) (Angueira et al., 2015; Guasch-Ferre et al., 2016). Studies have shown that high levels of branched-chain AA in early pregnancy are associated with an increased risk of GDM and maternal insulin resistance (Liu et al., 2020; Li et al., 2022). In addition, phenylalanine and its initial catabolism product were shown to be associated with maternal insulin resistance (Huhtala et al., 2018; Liu et al., 2020). Metabolites can regulate insulin sensitivity directly by modulating components of the insulin signaling pathway and indirectly by altering the flux of substrates through multiple metabolic pathways, including protein synthesis and degradation, hepatic gluconeogenesis, lipogenesis, and lipid oxidation (Yang et al., 2018). To date, few studies have investigated the dynamic changes in metabolites in different trimesters, and most metabolomics studies have focused on a single trimester cross-section. Therefore, longitudinal studies are essential to reveal the underlying pathophysiology of GDM development.

In this study, we performed both cross-sectional and longitudinal analyses to identify GDM-associated metabolites. Among the differentially expressed metabolites, cysteine (Cys) reduction was closely associated with GDM progression in pregnant women, and Cys supplementation alleviated GDM in female mice, suggesting a potential new strategy for GDM prevention and treatment.

## Results

### Study cohort

**Figure 1:**
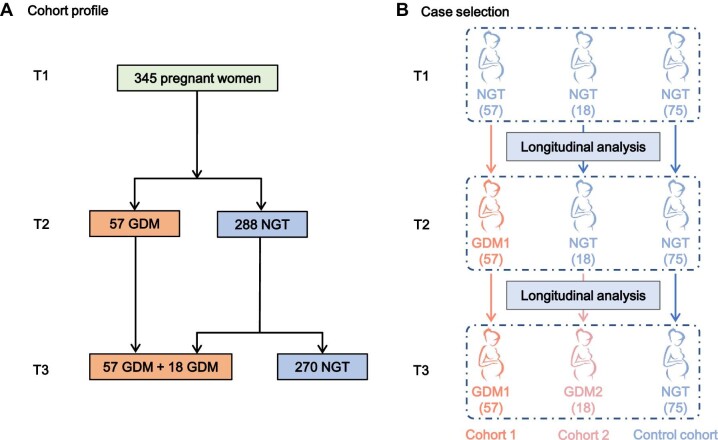
The study cohort. (**A**) In total, 345 pregnant women were recruited at the T1 visit. At the T2 visit, 57 women developed GDM, and 288 retained NGT. At the T3 visit, 18 more women developed GDM, and 270 retained NGT. (**B**) Cohort 1 included 57 pregnant women with NGT at the T1 visit who developed GDM at the T2 visit. Cohort 2 included 18 pregnant women with NGT at the T1 and T2 visits who developed GDM at the T3 visit. The control cohort included 75 pregnant women with NGT during all three trimesters. In total, 150 pregnant women were included in the analysis (pair-matched on pregestational BMI and age).

A total of 345 pregnant women without prepregnancy diabetes mellitus were enrolled in this study. They were visited at three time points, including the first trimester visit (9–13 gestational weeks, T1), the second trimester visit (24–28 gestational weeks, T2), and the third trimester visit (30–34 gestational weeks, T3) (Figure 1A). A total of 150 women were ultimately included in our nested case-control study. Cohort 1 included 57 pregnant women with NGT at the T1 visit who developed GDM at the T2 visit (referred to as GDM1 patients). Cohort 2 included 18 pregnant women with NGT at the T1 and T2 visits who developed GDM at the T3 visit (referred to as GDM2 patients). The control cohort included 75 pregnant women with NGT during all three trimesters. In the following study, cross-sectional analyses were performed to identify metabolic differences among different cohorts in the indicated trimester, while longitudinal analyses were performed within the same cohort to identify metabolic differences during GDM progression (from the T1 visit to the T3 visit) (Figure 1B; Lai et al., 2020). The GDM patients and NGT controls were pair-matched for pregestational body mass index (BMI) and age.

### Demographic and metabolic characteristics of the pregnant women included in the analysis

The demographic and metabolic characteristics are summarized in Table 1. Among the three cohorts, there were no significant differences in glucose levels, lipid levels, or prenatal characteristics, including age, prepregnancy BMI, and family history of diabetes at the T1 visit. At the T2 visit, cohort 1 had significantly higher levels of fasting plasma glucose (FPG), 1-h plasma glucose (1hPG), 2-h plasma glucose (2hPG), fasting insulin (FIns), 1-h insulin (1hIns), 2-h insulin (2hIns), homeostasis model assessment for β-cell function (HOMA-β), and homeostasis model assessment for insulin resistance index (HOMA-IR) than the control cohort (*P* < 0.05), while cohort 2 only had higher FPG levels than the control cohort (*P* < 0.05). At the T3 visit, both cohort 1 and cohort 2 had higher FPG levels than the control cohort (*P* < 0.05). Notably, 8.8% (5/57) of the GDM1 patients received insulin treatment until delivery, while 91.2% (52/57) of the GDM1 patients and all GDM2 patients received only lifestyle modification interventions to reach glycemic control.

**Table 1:** Demographic and metabolic characteristics of pregnant women in different trimesters among the three groups.

Variable	Control cohort (*n* = 75)	Cohort 1 (*n* = 57)	Cohort 2 (*n* = 18)	*P*-value
**Prenatal characteristics**				
Age (years)	31.08 ± 4.44	31.44 ± 4.66	29.94 ± 5.79	0.502
Prepregnancy BMI (kg/m^2^)	22.20 ± 2.28	22.24 ± 2.67	22.44 ± 2.64	0.933
Family history of diabetes, *n* (%)	4 (5.3)	4 (7.0)	0 (0)	0.513
**Baseline characteristics at the T1 visit**				
TC (mmol/L)	4.57 ± 0.74	4.59 ± 0.74	4.51 ± 0.60	0.920
TG (mmol/L)	1.42 ± 0.46	1.42 ± 0.43	1.42 ± 0.48	0.999
HDL-C (mmol/L)	1.69 ± 0.36	1.73 ± 0.58	1.47 ± 0.26	0.109
LDL-C (mmol/L)	2.19 ± 0.58	2.22 ± 0.73	2.22 ± 0.44	0.968
FPG (mmol/L)	4.51 ± 0.29	4.55 ± 0.39	4.64 ± 0.40	0.390
HbA1c (%)	5.18 ± 0.26	5.24 ± 0.23	5.14 ± 0.30	0.261
**Characteristics at the T2 visit**				
TC (mmol/L)	5.84 ± 1.11	5.93 ± 1.00	5.87 ± 1.05	0.897
TG (mmol/L)	2.28 ± 0.77	2.26 ± 0.68	2.30 ± 0.60	0.982
HDL-C (mmol/L)	1.87 ± 0.38	2.00 ± 0.37	1.72 ± 0.24	0.015
LDL-C (mmol/L)	2.92 ± 0.90	2.93 ± 0.79	3.09 ± 0.95	0.735
FPG (mmol/L)	4.41 ± 0.31	4.80 ± 0.50**	4.64 ± 0.28*	<0.001
1hPG (mmol/L)	7.13 ± 1.19	9.95 ± 1.41**	7.55 ± 1.30	<0.001
2hPG (mmol/L)	6.30 ± 0.90	8.24 ± 11.58**	6.34 ± 0.76	<0.001
HbA1c (%)	4.88 ± 0.32	4.92 ± 0.34	4.80 ± 0.41	0.460
GA (%)	12.61 ± 1.56	13.26 ± 1.49	12.72 ± 2.30	0.079
FIns (pmol/L)	48.88 ± 23.24	60.28 ± 31.08*	56.37 ± 47.55	0.095
1hIns (pmol/L)	364.59 ± 198.63	504.91 ± 310.08**	323.57 ± 185.77	0.002
2hIns (pmol/L)	309.97 ± 158.01	542.47 ± 424.52**	283.49 ± 251.28	<0.001
HOMA-β	114.03 (73.51–183.37)	228.84 (88.02–333.61)**	154.25 (78.00–237.87)	0.002
HOMA-IR	1.32 (0.97–1.75)	1.71 (0.98–2.56)*	1.30 (0.84–2.23)	0.042
**Characteristics at the T3 visit**				
TC (mmol/L)	6.34 ± 1.18	6.32 ± 1.03	6.29 ± 1.09	0.979
TG (mmol/L)	3.06 ± 0.93	3.04 ± 0.91	3.22 ± 0.78	0.751
HDL-C (mmol/L)	1.86 ± 0.37	1.91 ± 0.36	1.72 ± 0.29	0.139
LDL-C (mmol/L)	3.19 ± 0.98	3.03 ± 0.91	3.37 ± 0.88	0.368
FPG (mmol/L)	4.45 ± 0.29	4.65 ± 0.54**	5.27 ± 0.14**	<0.001
HbA1c (%)	5.19 ± 0.30	5.20 ± 0.36	5.41 ± 0.39	0.181

The data are expressed as mean ± SD or median (interquartile range). *P < 0.05 and **P < 0.01, compared to the control cohort.

### Cross-sectional analyses reveal the general metabolomic features of GDM

**Figure 2:**
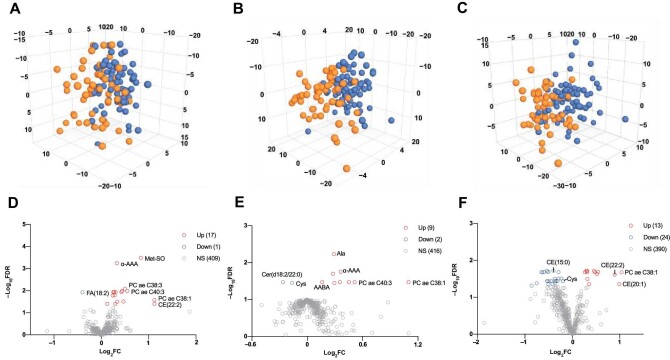
GDM-associated metabolites identified through cross-sectional analyses. (**A**–**C**) OPLS-DA score plots differentiate cohort 1 (orange) from the control cohort (blue) at the T1 (**A**), T2 (**B**), and T3 (**C**) visits. (**D**–**F**) Volcano plots show the differentially expressed metabolites in cohort 1 compared to the control cohort (FDR < 0.05) at the T1 (**D**), T2 (**E**), and T3 (**F**) visits. FC, fold change.

Cases in cohort 1 and the control cohort were separated in the orthogonal partial least squares discriminant analysis (OPLS-DA) score plots for all three trimesters, which indicated metabolic differences between the GDM1 group and the NGT group (Figure 2A–C). By comparing cohort 1 with the control cohort, we identified 18 metabolites significantly differentially expressed (false discovery rate [FDR] < 0.05) at the T1 visit, including 17 upregulated and 1 downregulated (Figure 2D), 11 differentially expressed metabolites at the T2 visit, including 9 upregulated and 2 downregulated (Figure 2E), and 37 differentially expressed metabolites at the T3 visit, including 13 upregulated and 24 downregulated (Figure 2F). Despite the fewest differentially expressed metabolites being identified at the T2 visit, the metabolic profiles of the GDM1 and NGT groups were separated more obviously at the T2 visit than at the T1 or T3 visit (Figure 2A–C), possibly due to more marked changes in these 11 differentially expressed metabolites.

Our results showed that the serum levels of α-aminoadipic acid (α-AAA) and asymmetric dimethylarginine (ADMA) increased in GDM patients (Supplementary Tables S1–S3), consistent with the findings of previous studies (Huynh et al., 2014; Guasch-Ferre et al., 2016). In addition, we identified some rarely reported GDM-related metabolites, such as aminobutyric acid (AABA). Furthermore, some metabolites were identified differentially expressed at only one time point, such as methionine sulfoxide (Met-SO) at T1, ADMA at T2, and CE(15:0) at T3 (FDR < 0.05), while some were differentially expressed at multiple time points, such as AABA at T2 and T3, α-AAA at T1 and T2, and Cys at T2 and T3 (FDR < 0.05). In conclusion, there are dynamic changes in metabolites during the course of GDM. To investigate the roles of metabolites in GDM progression, we need to further summarize their dynamic changes across different trimesters of GDM.

### Longitudinal analyses reveal metabolomic alterations associated with GDM progression

**Figure 3:**
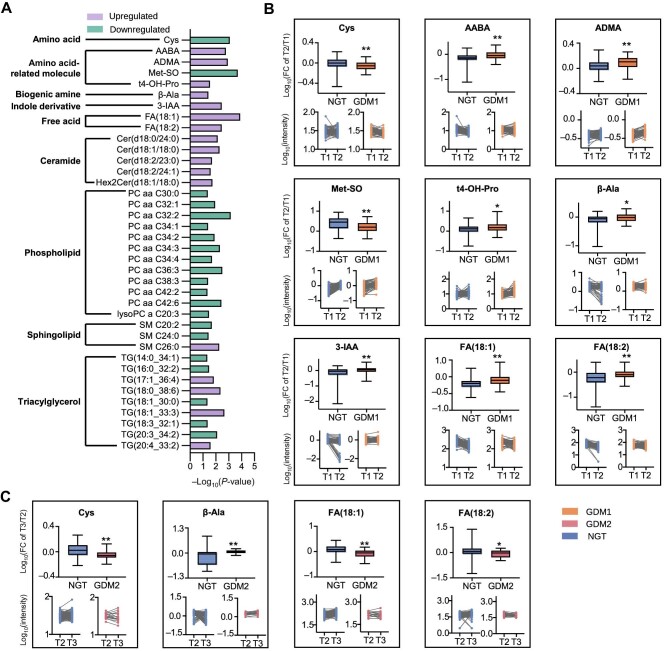
Alterations in GDM-associated metabolites identified through longitudinal analyses. (**A**) Thirty-eight differentially expressed metabolites associated with the progression of GDM (T2 vs. T1), including 17 upregulated (purple) and 21 downregulated (green) metabolites. (**B**) Fold changes (upper panels) and trajectories (lower panels) of nine metabolites in all individuals of cohort 1 from the T1 visit to the T2 visit. (**C**) Fold changes (upper panels) and trajectories (lower panels) of four metabolites in all individuals of cohort 2 from the T2 visit to the T3 visit. *P*-values were calculated using two-sided Mann–Whitney test. *P < 0.05; **P < 0.01.

The cross-sectional metabolomics study merely provides a general snapshot of GDM, while metabolome changes over time are more reflective of disease progression. Consequently, longitudinal changes in metabolites were tracked within each individual. We found that the levels of 38 metabolites in cohort 1 were significantly changed (*P* < 0.05) at the T2 visit compared with those at the T1 visit, 17 upregulated and 21 downregulated (Figure 3A). The 38 GDM progression-associated metabolites included 1 AA, 4 AA-related molecules, 1 biogenic amine, 1 indole derivative, 2 fatty acids (FAs), 5 ceramides (Cers), 12 PCs, 3 SMs, and 9 TGs (Figure 3A). Then, we focused on nine small-molecule metabolites with definite structural characteristics. Metabolite concentrations over time (trajectories) were tracked for each individual (Figure 3B; Supplementary Figure S2). The levels of Cys and Met-SO were significantly lower at the T2 visit than at the T1 visit (*P* < 0.05). In contrast, the levels of AABA, ADMA, 4-hydroxyproline (t4-OH-Pro), β-alanine (β-Ala), 3-indoleacetic acid (3-IAA), and FAs at the T2 visit were significantly higher than those at the T1 visit (*P* < 0.05).

Furthermore, longitudinal changes in metabolites from the T2 visit to the T3 visit were tracked in all individuals of cohort 2 who had a relatively late GDM onset. We found that the levels of Cys, PCs, and TGs were lower at the T2 visit than at the T3 visit, while the levels of β-Ala were significantly higher at the T2 visit than at the T3 visit (Figure 3C; Supplementary Figure S3). Notably, some metabolite trajectories were significantly different between GDM1 and GDM2 patients. In contrast to GDM1 patients, who had increased levels of FAs and Cers, GDM2 patients had reduced levels of FAs and Cers.

### Correlation-based integrative analysis reveals that Cys reduction has a significant impact on GDM progression

**Figure 4:**
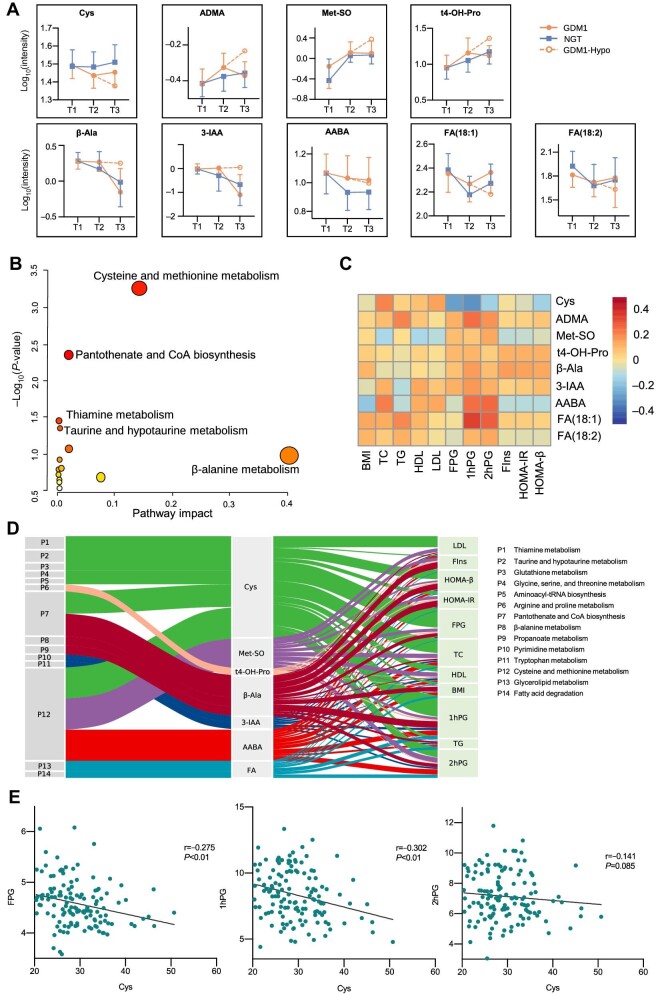
Correlation-based integrative analysis in cohort 1. (**A**) Dynamic changes in metabolites were traced in all three trimesters. GDM1-Hypo (dotted line) describes the subsequent metabolite trajectory continuing the trend from the T1 visit to the T2 visit in the GDM1 group. (**B**) Biological pathways associated with GDM onset. (**C**) Correlation analysis between clinical characteristics and differentially expressed metabolites was performed using the Spearman method at the T2 visit. Red boxes indicate positive correlations, while blue boxes indicate negative correlations. (**D**) Sankey plot showing the correlations among biological pathways, differentially expressed metabolites, and clinical characteristics at the T2 visit. The width of the ribbons between biological pathways and metabolites reflects the value of the significant difference [−log_10_(*P*-value)], while the width of the ribbons between metabolites and clinical characteristics reflects the value of correlation coefficient (r). (**E**) Correlation analysis between the levels of Cys and glucose was performed using the Spearman method at the T2 visit.

To further assess the dynamic changes in metabolites during GDM progression, we analysed the variation trends of the nine metabolites shown in Figure 3B throughout the three trimesters (Figure 4A; Supplementary Figure S4), using GDM1-Hypo to describe the subsequent metabolite trajectory that continued the trend from the T1 visit to the T2 visit in the GDM1 group. First, compared with that in the NGT group, the Cys concentration in the GDM1 group obviously decreased from the T1 visit to the T2 visit. After lifestyle modification or insulin treatment, although the Cys level in the GDM1 group was still lower than that in the NGT group, its downward trend slowed. Second, the upward trends of ADMA, Met-SO, t4-OH-Pro, β-Ala, and 3-IAA in the GDM1 group changed after treatment, making their concentrations at the T3 visit comparable to or even lower than those in the NGT group. In addition, the trends of AABA, FA(18:1), and FA(18:2) in the GDM1 group paralleled those in the NGT group after treatment. Collectively, after lifestyle modification or insulin treatment, these metabolites could partially or completely return to normal levels.

Next, we sought to determine which metabolites play crucial roles in GDM progression. First, we performed Kyoto Encyclopedia of Genes and Genomes (KEGG) pathway analysis to predict the biological pathways associated with the nine metabolites. The following pathways were found significantly enriched (*P* < 0.05): cysteine and methionine metabolism, pantothenate and CoA biosynthesis, thiamine metabolism, and taurine and hypotaurine metabolism (Figure 4B). Then, correlations between the differentially expressed metabolites and clinical characteristics at the T2 visit were determined (Figure 4C). Cys was negatively correlated with glucose levels, while ADMA, AABA, FA(18:1), and FA(18:2) were positively correlated with glucose levels, suggesting that AA biosynthesis and metabolism are essential for GDM progression. Finally, a Sankey plot was created to summarize the correlations among metabolites, biological pathways, and clinical characteristics (Figure 4D). Cys was involved in the 7 out of 14 pathways and correlated with clinical characteristics, including FPG, 1hPG, and TC. Specifically, Cys was negatively correlated with glucose levels (Figure 4E).

### Cys inhibits hepatic gluconeogenesis in GDM mice

**Figure 5:**
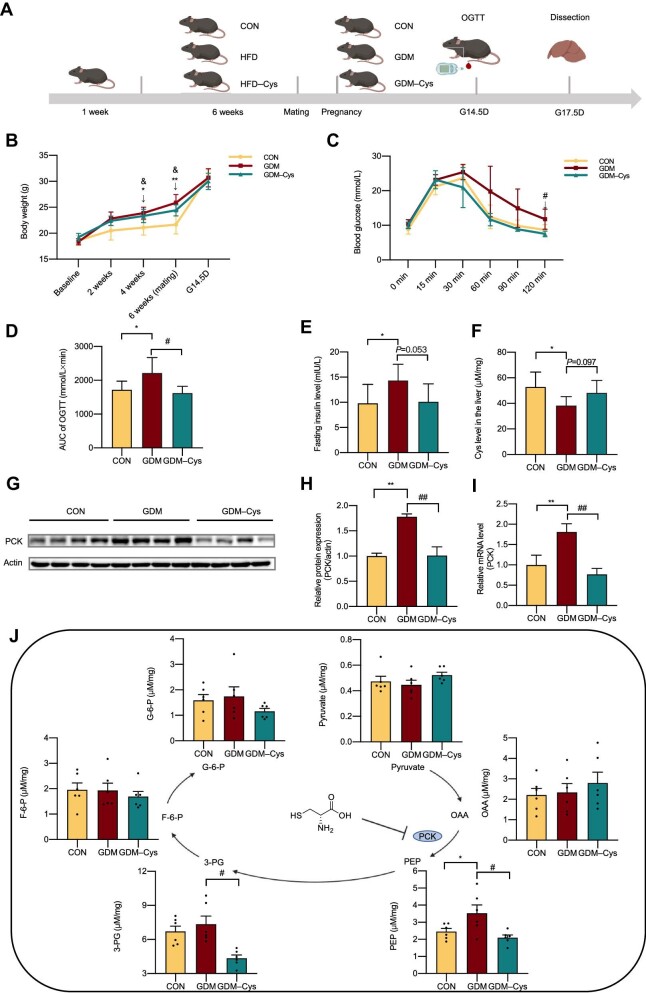
Cys supplementation inhibits hepatic gluconeogenesis in GDM mice. (**A**) The procedures for the *in vivo* experiments. (**B**) Body weight changes were detected in the CON, GDM, and GDM–Cys groups. (**C**) The OGTT was performed at G14.5D. Briefly, all mice received 2.0 g/kg glucose orally after fasting for 6 h; then, the glucose levels of the blood samples obtained from the tail vein at 0, 15, 30, 60, 90, and 120 min were determined using a glucose meter (*n* = 6). (**D**) The area under the curve (AUC) of the OGTT. (**E**) Fasting serum insulin levels were detected at G17.5D. (**F**) Liver Cys levels were detected at G17.5D. (**G**) Western blot analysis of PCK protein levels in the liver. (**H**) Quantification of PCK protein levels in **G**. (**I**) qRT-PCR analysis of PCK mRNA levels in the liver. (**J**) Metabolites involved in the hepatic gluconeogenesis pathway were detected. *P*-values were calculated using two-way ANOVA, followed by Tukey’s test (**B** and **C**), or one-way ANOVA (**D**–**J**). *P < 0.05 and **P < 0.01 for comparisons between the GDM and CON groups. ^&^*P* < 0.05 for comparisons between the GDM–Cys and CON groups. ^#^*P* < 0.05 and ^##^*P* < 0.05 for comparisons between the GDM–Cys and GDM groups.

To further investigate the effect of Cys on GDM, we used a high-fat diet (HFD)-induced hyperglycemic pregnancy mouse model, which closely mimicked human GDM (Figure 5A; also see Materials and methods). Before mating, the body weights of female mice fed a HFD or a HFD supplemented with Cys (the GDM or GDM–Cys group) significantly increased compared to those fed a standard diet (the CON group) (Figure 5B). On gestational day 14.5 (G14.5D), oral glucose tolerance tests (OGTTs) were performed. We found that the increased blood glucose levels and FIns levels induced by the HFD were ameliorated by Cys supplementation (Figure 5C–E). In addition, the decreased Cys levels in the liver induced by the HFD were restored by Cys supplementation (Figure 5F). Overall, hyperglycemia and increased insulin levels induced by HFD in GDM mice were alleviated by Cys supplementation.

Next, we examined the effects of Cys on hepatic glucose metabolism, including glucose transport, glycogen synthesis, and gluconeogenesis (Oosterveer and Schoonjans, 2014). As shown in Figure 5G–I, Cys supplementation reduced the protein and mRNA levels of phosphoenolpyruvate carboxykinase (PCK). In addition, Cys supplementation did not affect glucose transporter 2 (GLUT2), phosphorylated-glycogen synthase kinase-3β (p-GSK3β), or glucose-6-phosphatase (G-6-P) levels in the liver (Supplementary Figure S5). Thus, the hypoglycemic effect of Cys might be mediated via the inhibition of hepatic gluconeogenesis. Then, the concentrations of gluconeogenic metabolites in the liver, including pyruvate, oxaloacetate (OAA), phosphoenolpyruvate (PEP), 3-phosphoglycerate (3-PG), fructose-6-phosphate (F-6-P), and G-6-P, were examined. As shown in Figure 5J, the increased levels of PEP and 3-PG induced by the HFD were restored by Cys supplementation. Taken together, these findings suggest that Cys reduces GDM-induced hepatic gluconeogenesis by inhibiting PCK, which is an important factor in the pathogenesis of GDM (Plows et al., 2018).

## Discussion

GDM is a common complication leading to adverse pregnancy outcomes for both mothers and fetuses; however, its pathophysiology is only partially understood. Recent studies have highlighted metabolomics as a powerful tool for identifying potential biomarkers of GDM (Huynh et al., 2014). Here, we found that patients with GDM had significant longitudinal metabolomic changes compared with women with NGT and the GDM onset might be associated with specific metabolites. In addition, comprehensive management (e.g. lifestyle modification or insulin treatment) restored several metabolites to their normal levels and recovered metabolic homeostasis. Importantly, our study showed that Cys levels were decreased in women with GDM and Cys supplementation alleviated glucose metabolic disorders during pregnancy.

To date, several cross-sectional studies have assessed various metabolic signatures between women with GDM and women with NGT in different pregnancy periods (Bentley-Lewis et al., 2015; Alesi et al., 2021; Zhang and Yang, 2022). In our cross-sectional analysis, several identified differentially expressed metabolites were consistent with those from previous research, such as α-AAA, Met-SO, etc. (Wang et al., 2013; Gelaye et al., 2019). Some of these metabolites differed between women with GDM and women with NGT only in one trimester, while others differed significantly across multiple trimesters. Therefore, it is crucial to elucidate their dynamic changes during GDM progression and pathogenesis.

Through longitudinal studies, we observed dynamic changes in metabolites from normoglycemia to GDM and identified key metabolites that precede clinical phenotypes. We found that 38 metabolites were significantly dysregulated during GDM progression from T1 to T2. Among them, Cys was almost unchanged from T1 to T2 in the NGT group, while it decreased significantly in the GDM group during T2 and T3. Furthermore, correlation-based integrative analysis showed a significant impact of Cys on GDM progression.

Cys is a semiessential AA that can be obtained from the diet and produced from Met degradation via the trans-sulfuration pathway. Cys is a source of the synthesis of coenzyme A, glutathione (GSH), taurine, and H_2_S (Rehman et al., 2020). Previous studies have investigated Cys levels in association with glucose homeostasis. Patients with uncontrolled T2DM exhibit decreased blood levels of Cys, glycine, and GSH, and dietary supplementation with Cys and glycine can restore GSH synthesis and reduce oxidative stress and oxidative damage in the face of persistent hyperglycemia (Sekhar et al., 2011). In addition, Cys supplementation decreased glucose and hemoglobin A1c (HbA1c) levels in the blood, reduced oxidative stress, and inhibited NF-κB activation and insulin resistance in the livers of diabetic rats (Jain et al., 2009; Salman et al., 2013). Another study demonstrated that Cys increased cellular phosphatidylinositol-3,4,5-trisphosphate and glucose metabolism in 3T3L1 adipocytes (Manna and Jain, 2013). Therefore, Cys supplementation is beneficial for reducing inflammation and oxidative stress and has hypoglycemic effects. However, how Cys ameliorates glucose metabolic disorders remains largely unknown.

Hou et al. (2018) performed metabolomics analysis in 131 patients with GDM and 138 subjects with NGT only during the OGTT period and found that Cys levels were significantly lower in patients with GDM than in subjects with NGT, which was consistent with our findings. To further understand the pathophysiological roles of Cys in GDM, we established a GDM mouse model. Cys supplementation was found to alleviate GDM-related hyperglycemia, which was partially consistent with previous reports (Jain et al., 2009; Salman et al., 2013). Our results showed that Cys restrained the mRNA and protein expression of the key gluconeogenic enzyme PCK in the livers of GDM mice. Gluconeogenesis plays an essential role in maintaining glucose homeostasis, and PCK regulates the first rate-limiting step in hepatic gluconeogenesis, which catalyzes the conversion of OAA to PEP (Yu et al., 2021). Our experimental findings extend previous work by demonstrating that the hypoglycemic effect of Cys is related to the suppression of hepatic gluconeogenesis via PCK. The aminoacyl tRNA synthase enzyme can sense the concentration of AA in cells by recognizing aminoacylation modifications of lysine residues on the corresponding protein (He et al., 2018). Cysteinyl-tRNA synthetase senses the absence of Cys and activates adenosine monophosphate-activated protein kinase (AMPK) (Yuan et al., 2021). AMPK signaling induction is an ideal strategy for reducing insulin resistance, improving glucose and lipid metabolism, and ameliorating diabetes complications (Entezari et al., 2022). Cys, an AA with glucose metabolism-regulating properties validated in GDM animal models, might be a novel, effective, and safe treatment strategy for GDM. Further study regarding the detailed mechanism of Cys-mediated glucose regulation is needed.

Our current study had several strengths. First, we performed longitudinal analyses covering all three trimesters, allowing us to investigate potential metabolic changes in GDM progression. Second, we identified Cys in association with GDM progression and verified its role in GDM development through *in vivo* experiments. However, there were still some limitations. Although we analysed the metabolic profiles throughout pregnancy, due to the limited sample size, we did not conduct training and validation analyses. In addition, this was an observational study from a single center, and the enrolled subjects did not represent a random sample of pregnant Chinese women. Hence, further external validation is warranted, and the extrapolation of the results to different populations should be done with caution.

In conclusion, our results indicate that women with GDM begin to develop dysmetabolism during the transition from NGT to new-onset GDM. The dynamic changes in metabolites over the three trimesters reveal metabolite trajectory maps in GDM progression. In particular, Cys has been shown to play a crucial role in GDM development, and the hypoglycemic effect of Cys is related to the suppression of hepatic gluconeogenesis via PCK. Therefore, supplementation with Cys may be a new strategy for GDM prevention and therapy.

## Materials and methods

### Subjects

Pregnant women were recruited from Shanghai General Hospital from September 2018 to May 2020. The major eligibility criteria included the following: aged 18–45 years, at 10–13 gestational weeks of pregnancy, and not using medications affecting glucose tolerance. Women with pregestational type 1 diabetes mellitus (T1DM) or T2DM (self-reported diabetes history) or abnormal glucose metabolism during early pregnancy (fasting glucose at the T1 visit ≥5.1 mmol/L) were excluded from the study. Women were diagnosed with GDM if one or more of the following plasma glucose values during the 75-g OGTT (T2 or T3 visit) were met or exceeded: 0 h, 5.1 mmol/L; 1 h, 10.0 mmol/L; and 2 h, 8.5 mmol/L (Metzger et al., 2010). In addition to a 75-g OGTT at the T2 visit, fasting lipids, including serum total cholesterol (TC), TG, high-density lipoprotein cholesterol (HDL-C), and low-density lipoprotein cholesterol (LDL-C), FPG, FIns, and HbA1c were detected at all three visits. Finally, a total of 345 pregnant women without prepregnancy diabetes mellitus were enrolled at the T1 visit. During the follow-up, 57 women (16.5%) developed incident GDM at the T2 visit (defined as the GDM1 group), and 18 women (5.2%) developed incident GDM at the T3 visit (defined as the GDM2 group). In our nested case-control study, we selected 75 cases with GDM and 75 cases with NGT (pair-matched on pregestational BMI and age). The fasting serum samples of the 150 participants at the T1, T2, and T3 visits were determined by targeted metabolomics analysis. The study protocol was approved by the ethics committee of Shanghai General Hospital, Shanghai Jiao Tong University School of Medicine.

### Measurement of clinical metabolic characteristics

Plasma glucose levels were measured enzymatically. FIns and serum lipid (TC, TG, HDL-C, and LDL-C) levels were determined by chemiluminescent assays. HbA1c and glycated albumin (GA) levels were determined by high-performance liquid chromatography (HPLC). HOMA-β and HOMA-IR were calculated to evaluate β-cell function and insulin resistance using the following formulas: HOMA-β = 20 × FIns (mU/ml)/(FPG [mmol/L] − 3.5) and HOMA-IR = FPG (mmol/L) × FIns (mU/ml)/22.5 (Wallace et al., 2004).

### Metabolomic detection

The fasting serum samples collected from the 150 participants at the T1, T2, and T3 visits were evaluated via metabolomics. The MxP® Quant 500 kit was used to assay a total of 630 metabolites via mass spectrometry (MS)-based detection according to the manufacturer's protocol (Biocrates Life Sciences AG). Multiple reaction monitoring (MRM) in combination with the use of internal standards allowed for quantification of the metabolites using Biocrates’ MetIDQ™ software included with the kit.

### Animal experiment

All animal care and experimental procedures were conducted in accordance with the approval and guidelines of the Laboratory Animal Ethics Committee of Shanghai Institute of Materia Medica, Chinese Academy of Sciences (approval number: IACUC-2021-10-YY-19). In total, 30 female and 15 male C57BL/6J mice aged 6 weeks were purchased from Shanghai SLAC Laboratory Animal Co., Ltd. All mice were given adaptive feeding for 1 week under specific pathogen-free conditions. Then, the female mice were randomly divided into three groups (10 in each group). In the CON group, the mice were fed a standard diet and administered with 0.9% NaCl as a placebo by gavage once a day. In the HFD group, the mice were fed a HFD (60% kcal fat, 20% kcal carbohydrate, and 20% kcal protein; Dyets Inc.) and administered with 0.9% NaCl as a placebo by gavage once a day. In the HFD–Cys group, mice were fed a HFD (60% kcal fat, 20% kcal carbohydrate, and 20% kcal protein; Dyets Inc.) and administered with Cys (300 mg/kg dispersed well in 0.9% NaCl solution) by gavage once a day (Salman et al., 2013). All the male mice were fed a standard diet. After 6 weeks of individual diet feeding, all female mice were mated with male mice at a ratio of 2:1 in the same cage, and the presence of sperm or vaginal plugs was deemed G0.5D. The body weights of the female mice were measured every 2 weeks during the experiments.

On G14.5D, the female mice underwent an OGTT. The mice were fasted for 16 h before measurement, and drinking water was freely available. The tails were cut, and the blood glucose levels were detected at 0 min using a blood glucose meter (Abbott Diabetes Care Inc.). Then, glucose (2 g/kg) was administered by gavage, and the blood glucose levels were detected at 15, 30, 60, 90, and 120 min. Fasting blood was obtained from the ocular orbit of the mice at G17.5D. Serum insulin levels were measured using an insulin ELISA kit (Signalway Antibody).

Liver tissues were collected after the mice were sacrificed. The tissues were stored at −80°C until analysis. Tissue proteins were extracted with protein extraction reagents for western blotting experiments. Antibodies against PCK (Proteintech), G6PC (Proteintech), p-GSK3β(S9) (Abcam), GSK3β (Abcam), GLUT2 (Proteintech), and actin (Sigma) were used. Tissue RNA was isolated using TRIzol reagent (Invitrogen). The relative mRNA abundances of PCK were assessed by quantitative real-time polymerase chain reaction (qRT-PCR).

To quantify the gluconeogenic metabolite concentrations, an LC–MS/MS system with an UltiMate 3000 HPLC system (Thermo Scientific) and an LCMS-8050 triple quadrupole mass spectro-meter (Shimadzu) was used. The standards and samples were measured using MRM mode with optimal collision energies. The data were analysed using LabSolutions software.

### Data processing and statistical analysis

Metabolomics data from the 150 participants measured at the T1, T2, and T3 visits were processed. Individual metabolites with >40% missing values, i.e. measurements < the limit of detection (LOD), were excluded at any time point, which reduced the total number of analytes in the dataset from 630 to 427. For these 427 analytes, participants with values reported as ‘<LOD’ were imputed using the LOD/2 value for each specific analyte. OPLS-DA was conducted by SIMCA 14.1 (Umetrics AB). The FDR was acquired by correcting the *P*-value using the Benjamini–Hochberg method. The *P*-values of the longitudinal analysis were calculated using two-sided Mann–Whitney test. Pathway analyses were conducted on the basis of the KEGG pathway database on the platform MetaboAnalyst 5.0 (https://www.metaboanalyst.ca/).

For demographic and metabolic characteristics, the data are expressed as mean ± standard deviation (SD) for normally distributed variables and as the median with the interquartile range for skewed data. We conducted analysis of variance (ANOVA) or the Kruskal–Wallis test to determine differences among the three groups. Least significant difference tests were used to perform pairwise comparisons between two groups. The chi-square test was used for categorical variables. Moreover, we assessed the correlation between the metabolites and clinical characteristics using Spearman rank correlation coefficients. The *in vivo* experimental data were analysed using two-way ANOVA, followed by Tukey's test, or one-way ANOVA. Statistical significance was set at *P* < 0.05 or FDR <0.05. Statistical analyses were carried out using SPSS 26.0 (IBM Corp.).

## Supplementary Material

mjae010_Supplemental_File

## Data Availability

The raw metabolomics data are shown in Supplementary material. Other data from this study are available upon reasonable request.

## Notes

**Author contributions:** J. Liu and Y.W. had full access to all the data in the study and took responsibility for the data analyses. M.L., J. Li, J.Y., J. Liu, and Y.W. designed the study. M.L., J.Y., Y.G., Y.M., F.F., N.L., T.S., Y.P., and Y.W. conducted the clinical trial and enrolled and managed the patients. J.Y., Y.G., and Y.M. cleaned and analysed the clinical data. M.L., J. Li, Q.Z., and J. Liu performed the metabolomics study and analysed the data. M.L., J. Li, J.Y., Y.Z, J. Liu, and Y.W. designed and performed the *in vivo* experiments. M.L., J. Li, J.Y., J. Liu, and Y.W. discussed the data and wrote the manuscript.
