# Supplementary material for: Metabolomic profiling reveals decreased serum cysteine levels during gestational diabetes mellitus progression
Source: J Mol Cell Biol. 2024 Mar 1;16(3):mjae010. doi: 10.1093/jmcb/mjae010 (PMC11418039; doi:10.1093/jmcb/mjae010)
Supplement: mjae010_Supplemental_File [file mjae010_supplemental_file.pdf]

Supporting information for

Metabolomic profiling reveals decreased serum cysteine levels during gestational diabetes mellitus progression

Contents

Fig S1. Metabolites associated with GDM2 in different trimesters: the cross-sectional analysis.

Fig S2. Alterations of metabolites associated with GDM1: the longitudinal analysis

Fig S3. Alterations of metabolites associated with GDM2: the longitudinal analysis

Fig S4. Metabolites associated with GDM1 in different trimesters: the cross-sectional analysis.

Fig S5. Cysteine supplement in GDM mice.

Table S1. Significantly different metabolites in incident GDM1 vs NGT at T1.

Table S2. Significantly different metabolites in incident GDM1 vs NGT at T2.

Table S3. Significantly different metabolites in incident GDM1 vs NGT at T3.

Fig. S1: Metabolites associated with GDM2 in different trimesters: the cross-sectional analysis

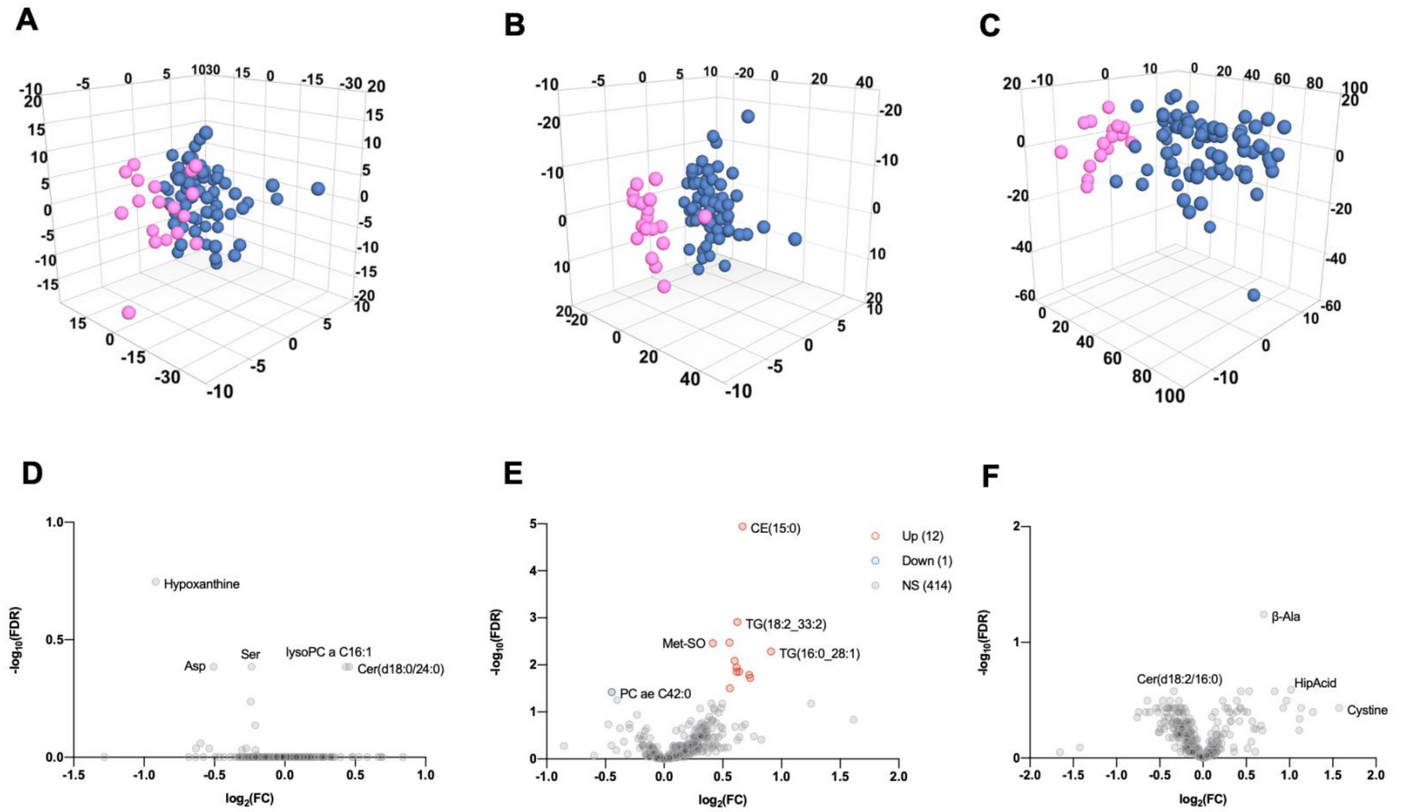

(A) Orthogonal partial least squares discriminant analysis (OPLS-DA) score plot was used to differentiate the cohort 2 patients (pink) from the control cohort (blue) at T1. (B) OPLS-DA score plot at T2. (C) OPLS-DA score plot at T3. (D) Volcano plot was used to show the differentially expressed metabolites between the cohort 2 patients and the control cohort (FDR<0.05) at T1. (E) Volcano plot at T2. (F) Volcano plot at T3.

Fig. S2: Alterations of metabolites associated with GDM1: the longitudinal analysis

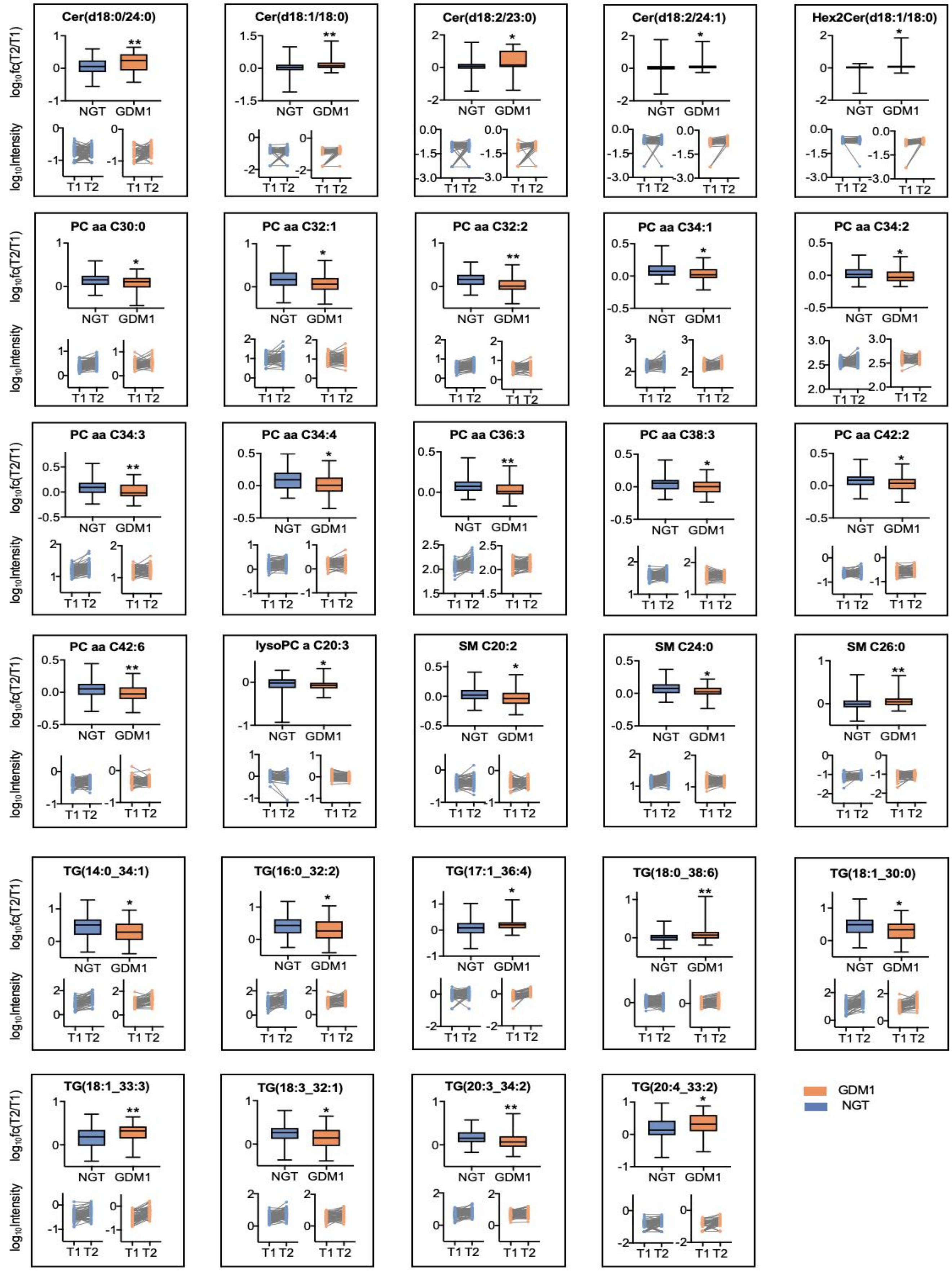

Fold changes (upper panels) and trajectory (lower panels) of metabolites within all individuals during GDM1 progression from T1 to T2.

Fig. S3: Alterations of metabolites associated with GDM2: the longitudinal analysis

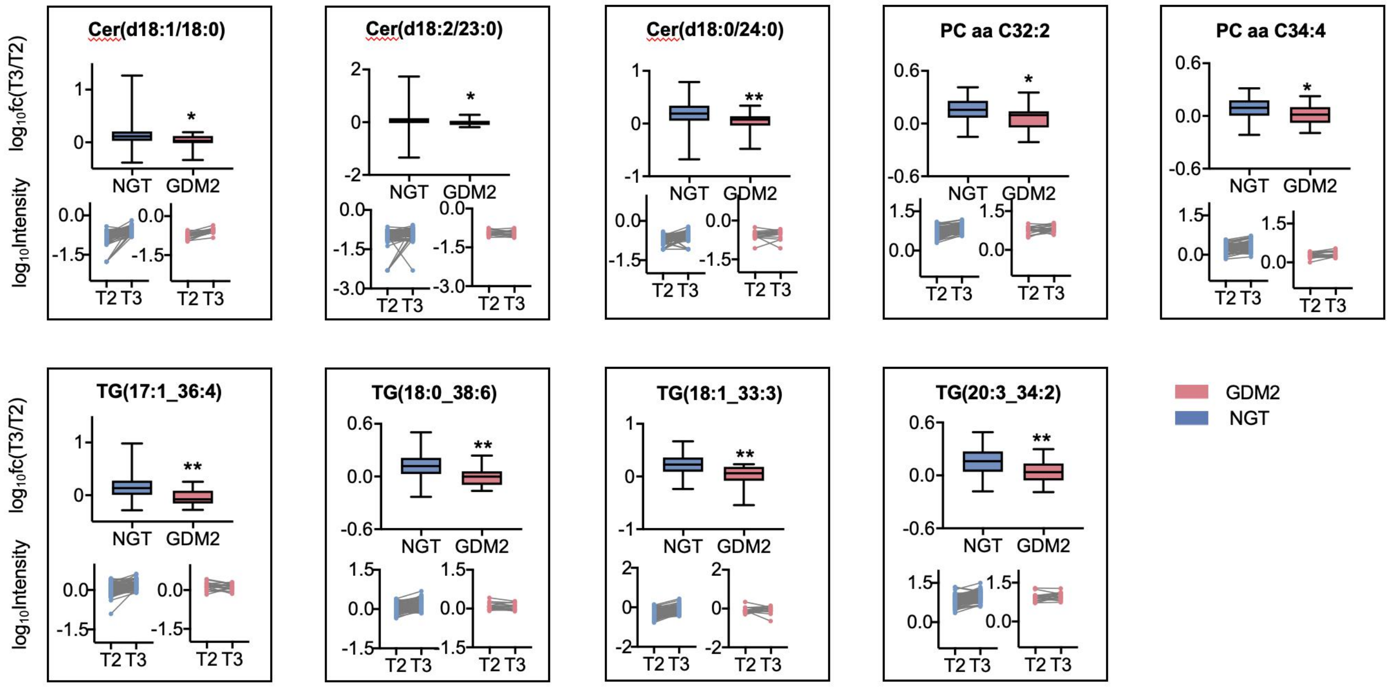

Fold changes (upper panels) and trajectory (lower panels) of metabolites within all individuals during GDM2 progression from T2 to T3.

Fig. S4: Metabolites associated with GDM1 in different trimesters: the cross-sectional analysis.

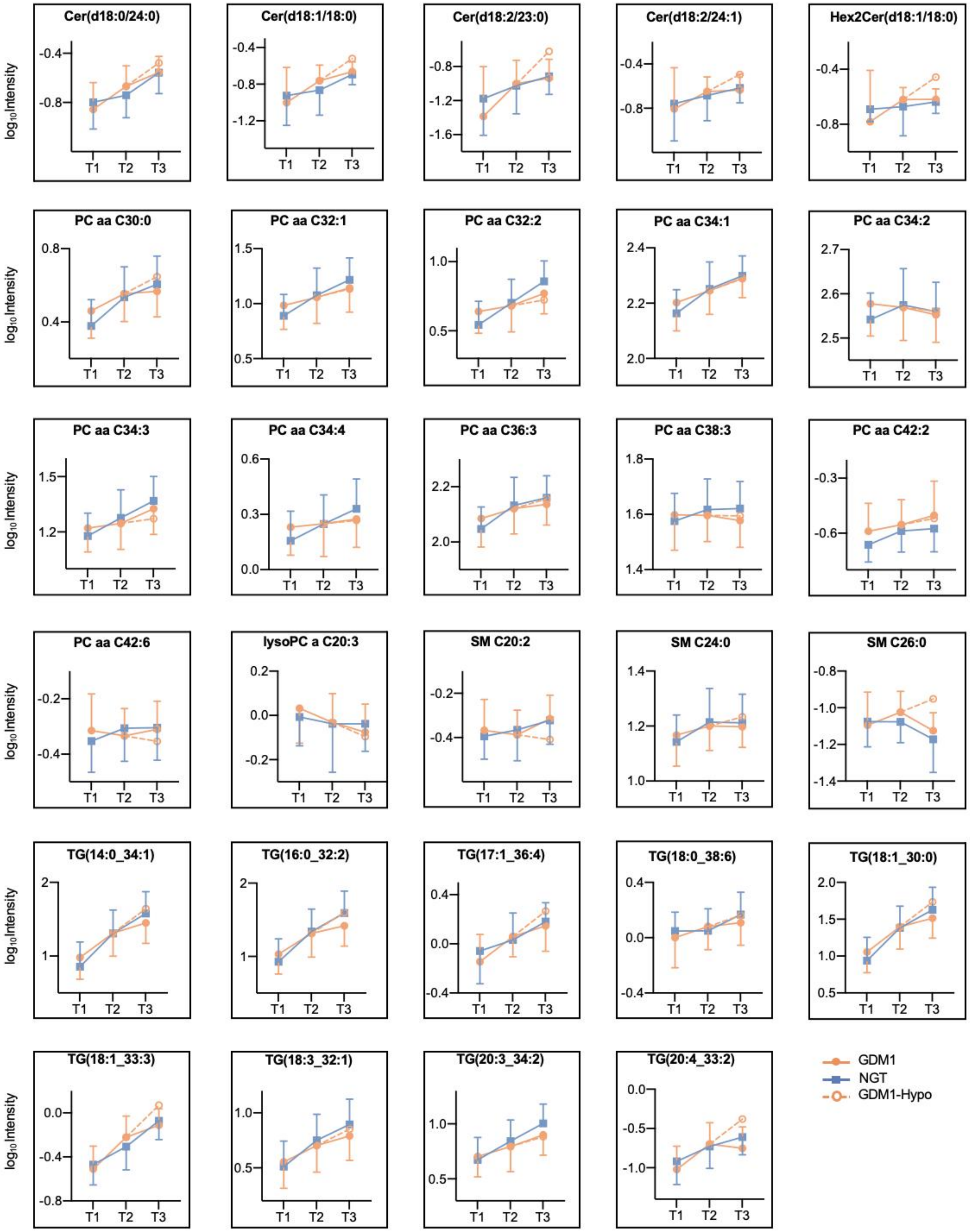

Metabolite dynamic changes were traced in the GDM1 and NGT groups during all three trimesters. GDM1-Hypo (dotted line) described the subsequent metabolite trajectory that continued the trend from the T1 visit to the T2 visit in the GDM1 group.

Fig. S5: Cysteine supplement in GDM mice.

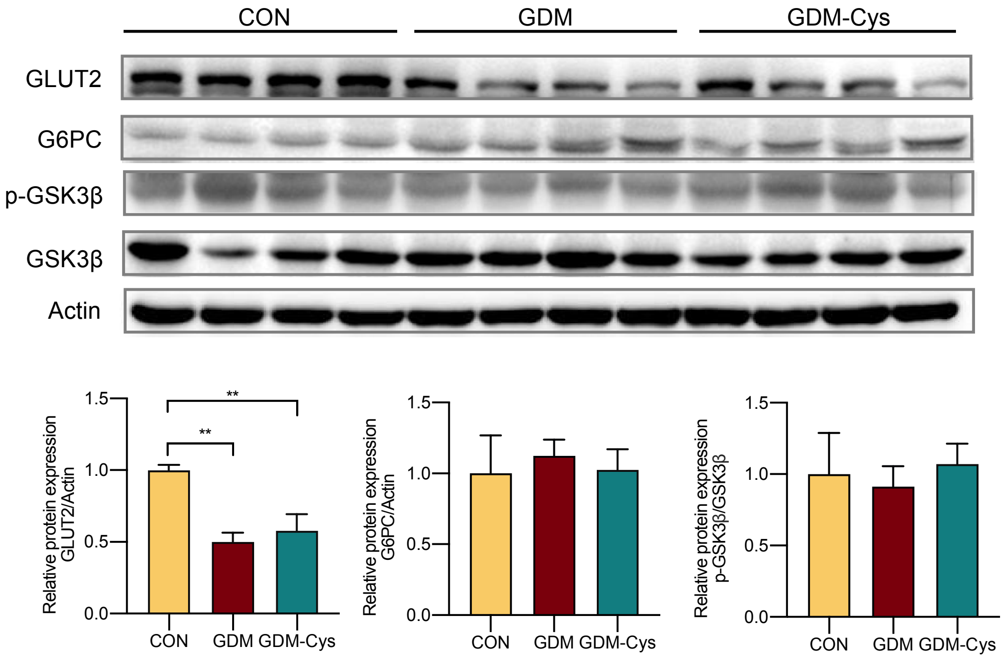

Western blot analysis and quantification of GLUT2, p-GSK3, GSK3β and G6PC expression levels of liver.

Data are presented as mean ± SD (n=4). ** *P*< 0.01 vs. CON group

Table S1. Significantly different metabolites in incident GDM1 vs NGT at T1.

No.	Metabolites	GDM1	NGT	FDR
1	α-AAA	0.94±0.22	0.75±0.23	5.64E-04
2	Met-SO	0.96±0.56	0.54±0.37	3.96E-02
3	CE(22:2)	0.52±0.72	0.24±0.19	1.18E-02
4	FA(18:2)	69.62±27.07	91.83±38.10	3.25E-04
5	PC aa C32:2	4.63±1.52	3.77±1.52	3.96E-02
6	PC aa C34:2	383.60±64.02	351.60±50.35	3.96E-02
7	PC aa C42:2	0.27±0.10	0.22±0.05	1.15E-02
8	PC ae C30:1	0.26±0.16	0.19±0.09	3.10E-02
9	PC ae C30:2	0.08±0.02	0.07±0.02	1.15E-02
10	PC ae C36:1	5.84±3.07	4.35±1.23	1.15E-02
11	PC ae C38:1	0.70±0.93	0.32±0.21	2.63E-02
12	PC ae C38:2	2.17±1.16	1.61±0.35	1.03E-02
13	PC ae C38:3	4.19±2.36	2.96±0.85	8.21E-03
14	PC ae C40:2	1.46±0.46	1.21±0.29	1.15E-02
15	PC ae C40:3	1.5±0.98	1.02±0.30	1.05E-02
16	PC ae C40:5	3.52±1.64	2.79±0.84	3.18E-02
17	PC ae C42:2	0.47±0.14	0.40±0.10	1.64E-02
18	PC ae C44:3	0.15±0.05	0.12±0.03	1.63E-02

Data are presented as the mean ± SD. Concentrations of metabolites are in μM.

Table S2. Significantly different metabolites in incident GDM1 vs NGT at T2.

No.	Metabolites	GDM1	NGT	FDR
1	AABA	11.51±4.47	8.95±2.71	1.77E-02
2	ADMA	0.48±0.09	0.43±0.08	3.38E-02
3	α-AAA	1.02±0.25	0.83±0.22	5.86E-03
4	Cys	27.66±4.74	31.01±6.21	3.38E-02
5	FA(18:1)	196.19±65.26	159.95±56.48	3.57E-02
6	Cer(d18:2/22:0)	0.24±0.08	0.29±0.07	3.57E-02
7	PC ae C36:1	6.86±3.08	5.37±1.81	3.38E-02
8	PC ae C38:1	0.87±1.06	0.41±0.39	3.38E-02
9	PC ae C38:3	5.10±3.00	3.73±1.36	3.38E-02
10	PC ae C40:2	1.69±0.49	1.39±0.38	2.00E-02
11	PC ae C40:3	1.84±1.19	1.29±0.46	3.38E-02

Data are presented as the mean ± SD. Concentrations of metabolites are in μM.

Table S3. Significantly different metabolites in incident GDM1 vs NGT at T3.

No.	Metabolites	GDM1	NGT	FDR
1	AABA	11.10±4.01	8.94±2.48	1.91E-02
2	CE(15:0)	4.6±1.94	6.07±2.09	1.91E-02
3	CE(20:1)	15.53±19.68	7.82±9.69	4.41E-02
4	CE(22:2)	1.40±1.54	0.75±0.54	2.45E-02
5	Cer(d18:1/24:0)	2.29±0.60	2.69±0.75	3.10E-02
6	Cer(d18:2/24:0)	0.42±0.14	0.51±0.16	2.09E-02
7	Cys	29.00±6.06	33.25±8.73	3.62E-02
8	DG(16:1_18:2)	1.12±0.44	1.52±0.90	3.62E-02
9	FA(18:1)	249.77±100.09	200.48±75.41	3.18E-02
10	PC aa C32:2	6.25±2.17	7.65±2.64	3.10E-02
11	PC ae C30:2	0.10±0.04	0.08±0.03	2.09E-02
12	PC ae C36:1	8.51±4.52	6.20±2.28	1.91E-02
13	PC ae C38:1	1.21±1.32	0.59±0.65	2.09E-02
14	PC ae C38:2	2.98±1.62	2.33±0.85	4.41E-02
15	PC ae C38:3	6.09±3.93	4.19±1.97	2.05E-02
16	PC ae C40:2	1.76±0.57	1.44±0.39	1.91E-02
17	PC ae C40:3	2.04±1.34	1.42±0.72	2.34E-02
18	PC ae C40:5	4.91±2.94	3.54±1.58	2.09E-02
19	ProBetaine	2.27±2.94	4.11±4.03	4.87E-02
20	SM C26:1	0.19±0.06	0.16±0.05	2.09E-02
21	TG(14:0_34:2)	27.53±16.23	39.60±25.96	3.70E-02
22	TG(14:0_34:3)	4.78±2.96	6.91±4.50	3.64E-02
23	TG(14:0_36:3)	37.07±14.49	47.73±22.57	3.62E-02
24	TG(14:0_36:4)	15.88±6.89	22.53±12.34	2.05E-02
25	TG(14:0_38:5)	0.89±0.52	1.25±0.76	3.70E-02
26	TG(16:0_30:2)	3.07±2.23	5.16±4.88	4.17E-02
27	TG(16:0_32:2)	31.66±20.49	48.98±33.45	2.09E-02
28	TG(18:1_32:2)	41.90±16.13	54.22±25.27	3.18E-02
29	TG(18:2_30:0)	26.73±14.78	40.29±26.75	2.09E-02
30	TG(18:2_30:1)	7.02±3.81	11.01±7.99	2.09E-02
31	TG(18:2_32:2)	28.07±12.11	39.89±21.67	2.05E-02
32	TG(18:2_33:2)	4.32±1.73	5.57±2.73	4.17E-02
33	TG(20:3_32:2)	0.66±0.34	0.95±0.47	1.91E-02
34	TG(20:3_34:2)	8.66±3.57	10.96±4.59	3.62E-02
35	TG(20:3_36:4)	1.33±0.53	1.65±0.62	3.62E-02
36	TG(20:4_32:2)	1.29±0.77	1.84±1.21	4.41E-02
37	TG(20:4_33:2)	0.21±0.11	0.28±0.15	4.41E-02

Data are presented as the mean ± SD. Concentrations of metabolites are in μM.
